# The effects and costs of an anti-bullying program (KiVa) in UK primary schools: a multicenter cluster randomized controlled trial – CORRIGENDUM

**DOI:** 10.1017/S0033291725000248

**Published:** 2025-04-03

**Authors:** Lucy Bowes, Malavika Babu, Julia R. Badger, Matthew R. Broome, Rebecca Cannings-John, Suzy Clarkson, Elinor Coulman, Rhiannon Tudor Edwards, Tamsin Ford, Richard P. Hastings, Rachel Hayes, Fiona Lugg-Widger, Eleri Owen-Jones, Paul Patterson, Jeremy Segrott, Mia Sydenham, Julia Townson, Richard C. Watkins, Holly Whiteley, Margiad E. Williams, the Stand Together Team, Judy Hutchings

An author’s name was incorrectly spelled in the article as originally published (Sadja Butt). This has been corrected to Sajda Butt in the HTML and PDF versions of the article.

The authors apologize for this error.
